# Subaortic Membrane With a Patent Ductus Arteriosus in an Adult Patient: A Challenging Diagnostic Dilemma

**DOI:** 10.7759/cureus.89625

**Published:** 2025-08-08

**Authors:** Ahmed Youssouf Addou, Jad Ait Lahmouch, Mohammed Aoudad, Amale Tazi, Aatif Benyass

**Affiliations:** 1 Cardiology, Cheikh Zaid International University Hospital, Rabat, MAR; 2 Cardiology, Abulcasis International University of Health Sciences, Rabat, MAR; 3 Cardiology, Faculty of Medicine and Pharmacy of Rabat, Mohammed V University, Rabat, MAR

**Keywords:** aortic aneurysm, aortic regurgitation, left ventricular outflow tract obstruction (lvoto), patent ductus arteriosus, subaortic membrane

## Abstract

Subaortic membrane (SAM) is a subtype of left ventricular outflow obstruction, rarely seen in adults. In some cases, SAM may be associated with other congenital defects. The association of patent ductus arteriosus (PDA) and SAM is the rarest, especially in adult patients. The management of these patients is delicate in terms of diagnosis, timing, and the appropriate surgical technique.

A 34-year-old patient with a known aortic heart murmur presented with exertional syncope. She was referred to our hospital for aortic valve replacement due to symptomatic aortic stenosis. Transthoracic and transesophageal echocardiography demonstrated a SAM with significant stenosis (mean gradient 48 mmHg) associated with a PDA, mild to moderate aortic regurgitation (AR), and an ascending aortic aneurysm. The heart team decided to proceed with membrane resection, aortic valve replacement, and ascending aortic graft.

This case highlights the challenge of managing adult congenital heart disease, particularly regarding diagnosis and optimal timing for surgical intervention. The subaortic membrane is a structural anomaly that can be either congenital or acquired. The primary indication for surgery is the symptomatic form or its association with severe AR. We believe that prophylactic surgery should be considered in isolated cases of mixed anomalies.

## Introduction

Subaortic stenosis (SAS) is a rare form of left ventricular outflow tract obstruction (LVOTO), accounting for 8-20% of cases [1]. It is caused by a fibrous curtain attached to the septum, resulting in localized LVOTO. Discrete subaortic membrane (SAM) represents the most common variety of SAS. In addition to congenital origins, these lesions can be acquired due to shear stress in the subaortic tract [2]. Guidelines are clear on managing symptomatic adult patients with significant SAS, but less clear on a prophylactic approach. Aortic regurgitation is also frequent, but there are no guidelines on the most suitable surgical approach, particularly in the context of an aortic aneurysm. We present a case, with a literature review, to explore the challenges of managing a SAM associated with aortic regurgitation, aneurysm, and patent ductus arteriosus in an adult patient.

## Case presentation

A 34-year-old African woman presented to the emergency department of an outside hospital with exertional syncope. She had no history of coronary disease, hypertension, diabetes, or dyslipidemia. Her past medical history included a heart murmur diagnosed at the age of 14 years, with no follow-up. She denied any previous diagnosis of rheumatic fever. The patient reported that during her last pregnancy, two years earlier, she experienced a gradual worsening of dyspnea, but she did not seek medical attention at the time. Six months prior to her syncope, she began experiencing worsening shortness of breath. An echocardiogram performed at the outside facility showed severe aortic stenosis. She was transferred to our hospital for aortic valve replacement.

Upon admission, physical examination revealed a 5/6 systolic aortic murmur along the left midsternal border radiating to the neck, accompanied by a diastolic murmur, with no peripheral signs. Her B-type natriuretic peptide value was 10 pg/mL. Other laboratory findings were normal (Table 1). ECG showed sinus rhythm with left ventricular hypertrophy and strain pattern.

**Table 1:** Laboratory finds at admission were normal AST: aspartate aminotransferase; ALT: alanine aminotransferase; INR: international normalized ratio; TSH: thyroid-stimulating hormone; BNP: B-type natriuretic peptide.

Test	Admission	Range
Hemoglobin (g/dL)	12.6	13.0-17.5
Leucocytes (x10^9^/L)	9.2	4.0-11.0
Platelets (x10^9^/L)	259	150-450
AST (U/L)	20	0-40
ALT (U/L)	13	0-41
Total bilirrubin (mg/dL)	1	<1.2
Direct bilirrubin (mg/dL)	0.7	<0.2
Prothrombin time (seconds)	12	11.6
INR	1.1	
Ferritin (ng/mL)	300	30-400
TSH (iU/mL)	1.7	0.30-4.20
BNP (pg/mL)	10	<35
Troponin (pg/mL)	2	< 14

**Figure 1:**
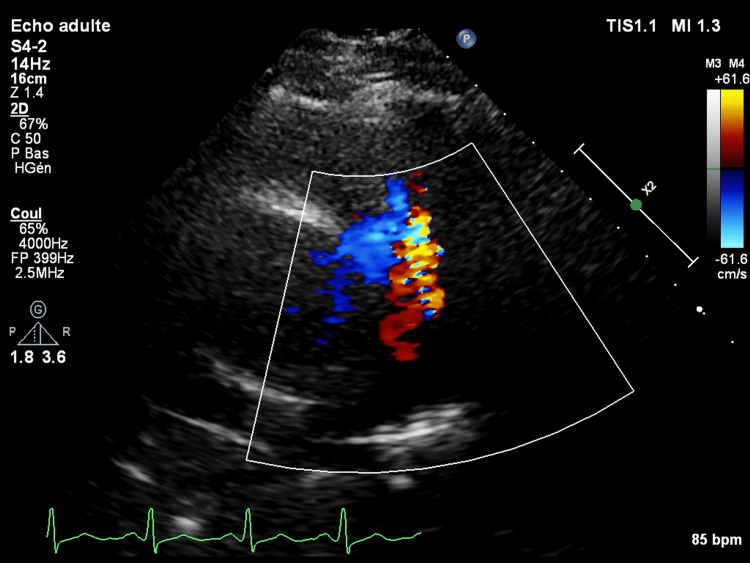
PSAX focus on pulmonary artery showing a PDA with left-to-right shunt PSAX: parasternal short-axis; PDA: patent ductus arteriosus.

The transthoracic echocardiography (TTE) on admission demonstrated eccentric left ventricular hypertrophy with a ventricular mass of 172 g/m², left ventricular end-systolic diameter of 22 mm/m², and an end-diastolic volume of 97 mL/m². Systolic function was preserved at 50% without wall-motion abnormalities. The parasternal long-axis view showed a subaortic membrane (SAM) located only 5 mm below the valve, with a maximum length of 8 mm. Significant flow acceleration in the left ventricular outflow tract (LVOT) was seen, with mean and maximal pressure gradients of 48 mmHg and 83 mmHg, respectively. The aortic valve was tricuspid with mild-to-moderate regurgitation of mixed mechanism, mild restriction of the non-coronary cusp, and a sino-tubular junction dilatation (19 mm/m²), defining a type Ia of the functional AR classification. The latter was due to a 23 mm/m² ascending aorta aneurysm. The aortic root was not dilated (13.5 mm/m²). The right ventricular function was normal, with a tricuspid annular plane systolic excursion of 19 mm and a fractional area change of 50%. TTE also showed a PDA of 4 mm with an exclusive left-to-right shunt and a gradient of 21 mmHg (Figure 1).

**Figure 2:**
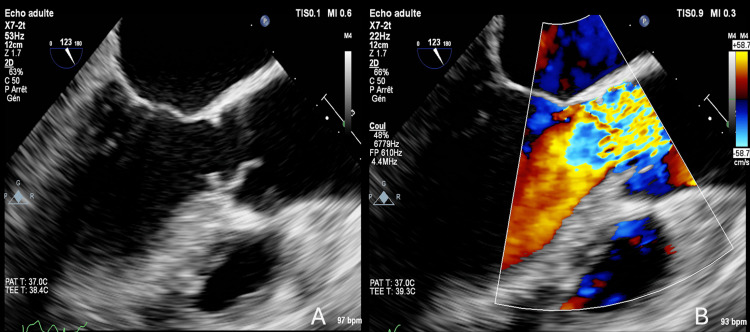
(A) Midesophageal long-axis view showing a discrete subaortic membrane in the LVOT, located 5 mm below the aortic valve. (B) Midesophageal long-axis view with color Doppler demonstrating obstruction in the LVOT. LVOT: left ventricular outflow tract.

A transesophageal echocardiography (TEE) was performed to better assess the aortic valve anatomy and the SAM. We found a circumferential SAM measuring 8 mm, located just below the aortic valve, creating an obstruction of the left ventricular outflow tract. The anatomy of the aortic valve was not compatible with aortic valvuloplasty, given the retraction of the non-coronary cusp with insufficient geometric height (10 mm) (Figure 2).

With respect to the patient’s preference, the heart team decided to perform a resection of the subaortic membrane and a mechanical aortic valve replacement. Because there was an ascending aortic aneurysm, a prosthetic tubular graft was placed. The PDA was clipped. The patient did not have any immediate complications and was discharged one week later in stable condition.

## Discussion

We have presented a case of discrete SAM associated with multiple anomalies: a PDA, mild-to-moderate aortic regurgitation, and an ascending aorta aneurysm. A discrete subaortic membrane is a subtype of LVOT obstruction with a prevalence of 6.5% in adults. It can be isolated or associated with other congenital defects. Other congenital anomalies include valvular or supravalvular stenosis as part of Shone disease and a ventricular septal defect. The combination of SAM and PDA is very rare, reported in only 5% of SAM cases [3].

When compared to other subaortic stenosis subtypes (fibromuscular ridge and tunnel stenosis), which have congenital origins, discrete SAM can be acquired (Figure 3). The steepness of the aorto-septal angle and morphological abnormalities of the LVOT (large aortic-mitral valve separation distance, narrow LVOT) may be risk factors for its development and progression [4].

Distinguishing SAM from other forms of LVOT obstruction, particularly hypertrophic obstructive cardiomyopathy, can be challenging [4]. When conventional TTE is equivocal, a multimodality approach combining TTE with TEE can reliably differentiate SAM, allowing a confident diagnosis, as in our case.

Adult patients with SAM may be asymptomatic for a long time, and symptoms are primarily related to the degree of obstruction. As reported in our case, dyspnea was first noted two years before the current presentation. SAM tends to progress slowly, with an LVOT gradient increase of 2.25 ± 4.7 mmHg per year [3].

The eccentric hypertrophy and subnormal systolic function found in our case have not been described in the literature before. Neither the insignificant left-to-right shunt nor the aortic regurgitation explains the ventricular dilatation. It is known that in late phases of aortic stenosis, left ventricular dilatation may be present with eccentric LV remodeling [5]. Therefore, SAM can cause an afterload mismatch if left untreated.

AR is another complication of SAM. It is reported in 63% of adult patients and is usually mild and non-progressive [6]. It is most often due to repetitive trauma of the aortic leaflets from the turbulent jet. A mixed mechanism was seen in our case, with both mild retraction of the non-coronary cusp and sinotubular junction dilatation secondary to the aneurysm.

Our patient was gravida 2, para 2, without miscarriage. In a cohort of 34 patients with asymptomatic SAM, pregnancy was associated with an elevation of the peak gradient by 25 mmHg, and more complications, such as miscarriage and preterm delivery, were noted. Fortunately, in our patient, pregnancy was not associated with complications [7]. Generally, asymptomatic women, or those with a mean LVOT gradient of less than 50 mmHg and preserved ejection fraction, will tolerate pregnancy [8]. Nevertheless, if a patient’s symptoms are resistant to medical therapy and she chooses to continue the pregnancy, surgery should be performed before labor and delivery.

Surgical therapy for SAM consists of membrane resection with or without myomectomy. The optimal timing for surgery, as suggested by European guidelines, is when the patient is symptomatic with a mean gradient ≥40 mmHg or severe AR [9]. Our case was a Class I recommendation for surgical treatment. But the question remains: is there an indication for prophylactic resection in cases with a mean gradient ≤40 mmHg or non-severe AR? The answer is unclear.

AR can progress postoperatively, as noted by Oliver et al. [3], with 5% of patients having moderate AR immediately after surgery and 10% progressing from no to moderate AR in eight years of follow-up. After surgery, the primary factor for recurrence and progression is a preoperative peak LVOT gradient ≥80 mmHg [10].

Another limitation of prophylactic surgery is the tendency for recurrence of SAM, seen in 11-16% of focal discrete SAM cases [11]. Risk factors for recurrence include female sex, age ≥30 years, preoperative gradient ≥80 mmHg, and inadequate resection [12].

Current European guidelines do not recommend antibiotic prophylaxis for either subvalvular stenosis or PDA, as they are considered intermediate risk for infective endocarditis. However, data on this issue are scarce. In our African context, these recommendations should be reconsidered [13].

## Conclusions

SAM represents a rare entity with a challenging diagnosis. Therefore, combining multimodal imaging (TTE, TEE, and cardiac computed tomography) is often necessary to confirm the diagnosis. Surgical treatment remains the gold standard, but with a non-negligible recurrence risk.
